# Molecular Characterization of Three Novel Large Deletions Causing α^0^-Thalassemia

**DOI:** 10.3390/ijms26188783

**Published:** 2025-09-09

**Authors:** Sara Ferrer-Benito, María Pilar Ricard Andrés, María José Murúzabal, Jorge M. Nieto, Fernando A. González, Belén Ortega-Montero, Ana Villegas, Celina Benavente, Paloma Ropero

**Affiliations:** 1 Servicio de Hematología, Hospital Clínico San Carlos, 28040 Madrid, Spain; saraferrerbenito@gmail.com (S.F.-B.); jorgemartineznieto@gmail.com (J.M.N.); fernandoataulfo.gonzalez@salud.madrid.org (F.A.G.); aortegam@salud.madrid.org (B.O.-M.); celinamaria.benavente@salud.madrid.org (C.B.); 2 Grupo de Investigación en Hematología, Hospital Clínico San Carlos, Instituto de Investigación Sanitaria Hospital Clínico San Carlos, 28040 Madrid, Spain; 3 Servicio de Hematología, Hospital Universitario Fundación Alcorcón, 28922 Madrid, Spain; mpilar.ricard@salud.madrid.org; 4 Servicio de Hematología, Hospital Universitario Marqués de Valdecilla, 39008 Santander, Spain; mariajose.muruzabal@scsalud.es; 5 Facultad de Medicina, Complutense University of Madrid, 28040 Madrid, Spain; anamaria.villegas@salud.madrid.org

**Keywords:** α-thalassemia, large deletions, MLPA, NGS, HBA1, HBA2, HS-40, Devyser, structural variants, diagnosis

## Abstract

Alpha-thalassemia is most often caused by large deletions within the α-globin gene cluster which reduce or abolish α-globin chain synthesis. Several common deletions are well described, but atypical structural variants remain underdiagnosed. In this study, we report three novel large heterozygous deletions of the α-globin cluster. The variants were identified in unrelated patients who presented with persistent microcytosis and hypochromia in the absence of iron deficiency or structural hemoglobin variants. A stepwise molecular diagnostic approach was applied. It combined commercial deletion screening assays, Sanger sequencing, Multiplex Ligation-dependent Probe Amplification (MLPA), and targeted Next-Generation Sequencing (NGS) with the Devyser Thalassemia panel. MLPA detected three deletions ranging from ~17 kb to ~360 kb. All involved the critical HS-40 regulatory region and both HBA1 and HBA2 structural genes, consistent with α^0^-thalassemia alleles. Next-Generation Sequencing confirmed the extent of each deletion and refined their genomic boundaries. Comparative genomic mapping showed that these deletions are distinct from classical variants such as --SEA or --MED, indicating novel structural configurations. Clinically, all patients displayed a carrier phenotype, with normal HbF levels (<1%) and normal or slightly reduced HbA2 values. This study broadens the mutational spectrum of α^0^-thalassemia and demonstrates the diagnostic value of combining MLPA and NGS in patients with unexplained microcytosis. By enabling accurate distinction from iron-deficiency anemia and other microcytic disorders, these findings have direct translational implications for improving diagnostic precision and genetic counseling in clinical practice.

## 1. Introduction

Alpha thalassemia is an inherited disorder of hemoglobin synthesis characterized by reduced or absent production of alpha-globin chains, leading to microcytic and hypochromic anemia [1,2]. The most frequent cause is large deletions in the α-globin gene cluster (HBA), located on chromosome 16p13.3, which includes the HBA1 and HBA2 genes [3]. These deletions account for 90–95% of cases described worldwide, while the remaining 5–10% are attributed to non-deletional mutations, such as promoter variants, nonsense mutations, splice site alterations, and insertions or duplications [2,4,5]. Globally, the prevalence of alpha thalassemia carriers varies significantly, reaching over 40% in certain regions of Southeast Asia, such as Thailand, southern China, and Malaysia [6,7,8]. In contrast, in non-endemic areas such as Europe, the overall prevalence is lower and is often underestimated due to clinical heterogeneity and the lack of systematic genetic screening programs [9,10]. Nevertheless, countries like Spain show a relevant prevalence, especially in regions with Mediterranean ancestry and among migrant populations from areas of high endemicity, making alpha thalassemia a diagnosis that should be more frequently considered in clinical practice [11,12].

The clinical severity of alpha thalassemia depends on the number of functional genes affected. It ranges from silent carriers (a single deletion) to thalassemic traits (two genes) to moderate forms such as HbH disease (three affected genes) [13,14]. The most severe form is alpha thalassemia major (hydrops fetalis with hemoglobin Bart’s), caused by the complete deletion of all four alpha genes and associated with intrauterine or neonatal death without advanced prenatal interventions [15,16]. This condition is typically due to the inheritance of two α^0^-thal alleles (deletions affecting both HBA1 and HBA2) [17,18]. The most prevalent deletions responsible for α^0^-thalassemia include --SEA (Southeast Asia), --MED (Mediterranean), --FIL (Philippines), and --THAI (Thailand), all of which are well-characterized molecularly and have defined geographic distributions [19,20]. However, atypical structural deletions, which are larger or have non-canonical breakpoints, continue to be underdiagnosed [21,22,23]. Most of these variants escape detection by conventional techniques such as multiplex PCR or strip-hybridization assays, highlighting the need to implement more sensitive tools such as MLPA (Multiplex Ligation-dependent Probe Amplification) and NGS (Next-Generation Sequencing) in the diagnosis of hemoglobinopathies [24,25,26,27].

A fundamental aspect in the regulation of HBA gene expression is the HS-40 region (also called MCS-R2), a remote enhancer located upstream of HBA2 that coordinates the expression of both alpha genes through chromatin looping and interaction with specific erythroid transcription factors [28,29,30]. Functional studies have shown that deletion of HS-40 leads to a drastic reduction in transcription, even when the structural HBA1 and HBA2 genes remain intact [31,32]. In addition to HS-40, other key distal regulatory regions within the α-globin cluster have been described, collectively referred to as ranging from MCS-R1 to MCS-R4 (Multispecies Conserved Sequences) [33]. These sequences act as highly conserved erythroid enhancers and cooperate in the spatial and temporal regulation of HBA1 and HBA2 [34,35]. HS-40 (MCS-R2) is the main of these enhancers, but MCS-R1, MCS-R3, and MCS-R4 also participate in chromatin loop formation and in the binding of factors such as GATA1, NF-E2, and KLF1, which are essential for efficient transcription in erythroblasts [36,37,38,39]. Therefore, structural variants involving this region should be considered pathogenic and may contribute to the clinical phenotype even in heterozygosis [40,41].

In clinical settings such as Spain, where there is a mixture of populations with moderate to high risk of thalassemia, the finding of persistent microcytosis without iron deficiency or structural hemoglobin variants poses a diagnostic challenge [18,33]. In this context, the identification of atypical deletions of the α-cluster by advanced molecular methods is essential, both for individual diagnosis and for genetic counseling and the prevention of hydrops fetalis cases [12,22,34]. In this study, we describe, for the first time, three previously unreported structural deletions affecting the α-globin cluster. These variants were characterized by MLPA and targeted NGS, which allowed for the precise genomic definition of each and confirmed the simultaneous loss of the HBA1, HBA2, and HS-40 genes [28,30,33]. Our findings expand the molecular spectrum of α^0^-thalassemia and underline the clinical utility of advanced molecular technologies in the diagnosis of non-phylogenetic microcytic anemias [26,29,37].

## 2. Results

All three patients showed a hematological profile consistent with microcytosis and hypochromia, with red blood cell counts at or above the upper limit of normal and no evidence of iron-deficiency anemia. Hemoglobin levels were within the normal or slightly reduced range (15.5 g/dL in Patient 1, 12.4 g/dL in Patient 2, and 10.8 g/dL in Patient 3). Mean corpuscular volume (MCV) was markedly decreased in all cases (69.0, 63.0, and 57.7 fL, respectively), as was the mean corpuscular hemoglobin (MCH: 18.0, 19.6, and 17.7 pg). These indices are consistent with a microcytic, hypochromic phenotype typical of α^0^-thalassemia carriers. In adults, MCH < 25 pg is considered a stable and reliable index for the suspicion of α^0^-thalassemia [13]. Hemoglobin fraction analysis confirmed normal or slightly reduced HbA2 (2.1–2.9%) and HbF (<1%), further supporting the carrier state. Detailed hematological data are summarized in Table 1.

Molecular testing ruled out common alpha-thalassemia deletion mutations using commercial strip assays (ViennaLab) and found no point mutations via Sanger sequencing. However, MLPA analysis revealed large heterozygous deletions in all three cases affecting key regions of the HBA locus, including the HS-40 regulatory region (MCSR2) and the structural genes HBZ, HBA2, and HBA1.

**Figure 1:**
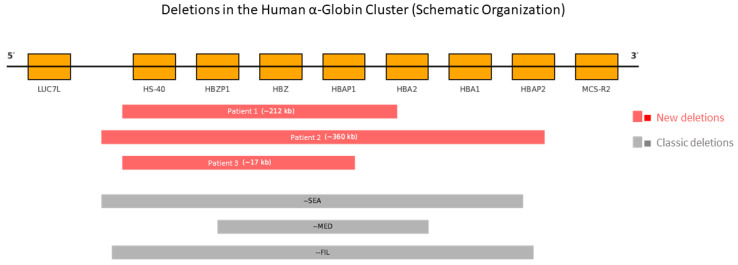
Schematic representation of the human α-globin cluster (16p13.3). Gene organization from 5′ to 3′ (from LUC7L to MCS-R2). The three newly identified deletions (in red), as detected by MLPA, are shown alongside the classic deletions --SEA, --MED, and --FIL (in gray). Each rectangle represents the genomic interval affected by the corresponding heterozygous deletion. Genomic positions are not to scale.

With the introduction of NGS, retrospective analysis of these samples using the Devyser Thalassemia panel confirmed the MLPA-detected deletions, validating the loss of critical regions including HBA1, HBA2, and HS-40, reinforcing their classification as full α^0^ deletions. Patient 1: MLPA showed a heterozygous loss of probes 1 to 30 (approx. 212 kb), encompassing HBZ, HBA2, HBA1, and HS-40. Patient 2: MLPA revealed a heterozygous loss of probes 1 to 33 (approx. 360 kb), including the same elements. Patient 3: MLPA indicated a heterozygous loss of probes 1 to 29 (approx. 17 kb), again affecting HBZ, HBA2, HBA1, and HS-40. These findings are summarized in Table 2 and illustrated in Figure 1.

Compared to the classical α^0^-thalassemia deletions, the three variants identified here display distinct structural features. The --SEA (~20 kb), --MED (~30 kb), and --FIL (~45 kb) deletions are well characterized and consistently involve HBA2, HBA1, and HS-40. In contrast, Patient 1 carried a ~212 kb deletion and Patient 2 a ~360 kb deletion, both encompassing not only HBA2, HBA1, and HS-40, but also the upstream HBZ gene and flanking intergenic regions. Patient 3 harbored a ~17 kb deletion with atypical breakpoints that differ from any previously described variant. These findings confirm that the deletions extend beyond the canonical boundaries of the classical alleles and affect a broader genomic interval that includes both structural and regulatory elements of the α-globin cluster.

Targeted NGS confirmed the deletions detected by MLPA and refined their genomic extent based on read-depth coverage analysis. However, the exact nucleotide breakpoints were not sequenced directly. The boundaries were inferred from the loss of NGS coverage in combination with the absence of MLPA probes across the HBA1, HBA2, and HS-40 regions. Thus, the deletions could be mapped with high genomic resolution, but precise junction sequences remain to be determined.

All three deletions have been submitted to the HbVar and IthaGenes databases (submission ID pending), to facilitate future clinical and molecular reference.

## 3. Discussion

This study describes, for the first time, three new deletions responsible for α^0^-thalassemia, identified by MLPA and characterized at high resolution with an NGS panel specific for hemoglobinopathies [25,28]. The three variants share a common genomic pattern, with heterozygous loss of the structural genes HBA1, HBA2, and the regulatory region HS-40, justifying their classification as complete deletions of the α-globin cluster [29,30]. At the molecular level, the combined loss of HBA1 and HBA2 directly compromises α-chain synthesis, while deletion of HS-40 (MCS-R2), the main erythroid enhancer of the locus, leads to transcriptional abolition even in the presence of intact structural genes [32,35]. Functional studies have shown that deletion of this region has an impact comparable to that of complete structural deletions, both in cellular models and gene expression analyses in bone marrow [38,40]. This finding is clinically relevant, as variants affecting exclusively regulatory regions may go unnoticed in incomplete genetic analyses [41].

In addition to HS-40, other regulatory regions within the α-globin cluster have been characterized, known as MCS-R1, MCS-R3, and MCS-R4. These regions function as secondary erythroid enhancers and participate in chromatin loop formation that brings regulatory elements closer to the HBA promoter, facilitating efficient transcription in erythroblasts [34,36]. Structural alterations affecting these regions can have epigenetic and functional effects comparable to the deletion of HS-40, especially if multiple enhancer sequences are compromised [39,42].

In contrast to the classical α^0^-thalassemia deletions (--SEA ~20 kb, --MED ~30 kb, and --FIL ~45 kb), which are limited to the α-globin genes and the HS-40 enhancer, the variants described here extend further. Patient 1 (~212 kb) and Patient 2 (~360 kb) deletions encompassed not only HBA2, HBA1, and HS-40, but also the upstream HBZ gene and adjacent intergenic regions, whereas Patient 3 (~17 kb) involved atypical breakpoints that differ from any previously reported variant. These structural differences confirm that the deletions are novel and distinct from classical alleles, suggesting a more complex genomic architecture [3,7,9]. In particular, the ~360 kb deletion observed in Patient 2 exceeds the conventional boundaries of the HBA cluster, potentially disrupting intergenic regions or uncharacterized distal regulatory elements [24,28]. Such alterations could have additional consequences for local chromatin organization, nuclear folding, or transcriptional stability, although these effects require functional validation [31,43]. Previous studies have reported other rare deletions spanning both structural and regulatory elements of the α-globin cluster, such as a 107 kb deletion in the Chinese population or large rearrangements identified through third-generation sequencing [27,29,30]. Together, these findings reinforce the importance of considering atypical structural variants in the differential diagnosis of microcytic anemias [10,37].

From a clinical point of view, the three patients presented a phenotype compatible with heterozygous carriers of α^0^-thalassemia, i.e., microcytosis and hypochromia with normal total hemoglobin and no iron deficiency [18,20]. This profile, although typical, can easily be confused with other microcytic anemias, such as iron deficiency or mild forms of beta thalassemia [13]. Normal HbA2 and HbF fractions, both by HPLC and capillary electrophoresis, reinforce the importance of considering alpha thalassemia in cases of unexplained microcytosis, even in the absence of family history [14,34].

The diagnostic algorithm used in this study demonstrates the effectiveness of a stepwise strategy, starting with conventional techniques (screening for common deletions and Sanger sequencing) and continuing with MLPA as a key tool to detect large deletions. In our setting, multiplex PCR for common deletions is used as the first step because it remains cost-effective and widely available in Spain. MLPA is reserved for cases with persistent clinical suspicion when initial PCR and Sanger sequencing are negative Subsequent confirmation by targeted NGS not only verified the findings, but also refined the genomic intervals affected. Although exact nucleotide junctions were not sequenced, the combined use of MLPA and NGS allowed for accurate delineation of the deleted regions and provided useful information for databases such as HbVar or IthaGenes [4,5,37]. These results reinforce the need to include structural analysis methods of the HBA cluster in the diagnosis of microcytic anemias of unclear cause, especially in contexts where rare variants may be underdiagnosed [26,28,44].

**Figure 2:**
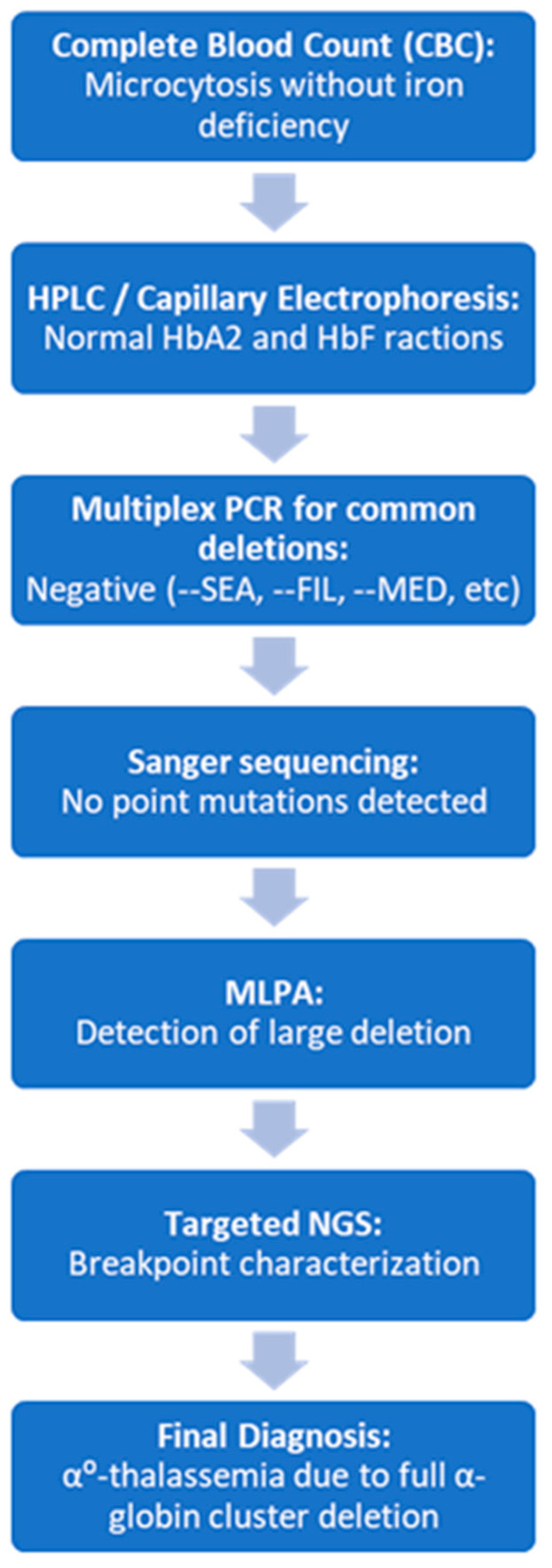
Diagnostic workflow for the identification of rare α^0^-thalassemia deletions. The stepwise algorithm begins with routine hematological and biochemical evaluation (including complete blood count and Hb analysis by HPLC or capillary electrophoresis). In cases of persistent microcytosis with normal iron status and no hemoglobin variants, standard molecular testing is performed for common deletions and point mutations (gap-PCR and Sanger sequencing). If negative, multiplex ligation-dependent probe amplification (MLPA) is applied to detect large deletions. Targeted next-generation sequencing (NGS) is then used to confirm and delineate the extent of structural variants. This strategy enables the identification of atypical deletions involving HBA1, HBA2, and regulatory elements such as HS-40, improving diagnostic accuracy and genetic counseling.

This stepwise approach has been synthesized into a diagnostic algorithm (Figure 2), which summarizes the clinical and molecular workflow followed in the analyzed cases. Its implementation may serve as a practical guide for centers facing persistent microcytosis without an apparent cause, facilitating the early diagnosis of rare forms of alpha-thalassemia.

In practice, only a minority of cases with unexplained microcytosis after PCR and MLPA proceed to targeted NGS. In our experience, fewer than 5% of suspected thalassemia cases require this step. The current cost of the Devyser Thalassemia NGS panel in Spain is approximately €250–300 per sample, which limits its use mainly to unresolved cases or research purposes.

Since 2003, Spain has had a universal neonatal screening program for hemoglobinopathies, driven by increased detection of sickle cell anemia in newborns, largely due to immigration from regions with high hemoglobinopathy prevalence [18,26,44]. This screening is part of national programs included in the pan-European consensus on neonatal detection, covering countries such as the United Kingdom, France, the Netherlands, Germany, and Spain, and is mainly focused on structural hemoglobinopathies such as HbS and beta thalassemia major [26,27,44]. However, alpha thalassemia, especially in its carrier form or associated with unusual structural variants, often escapes detection. Therefore, in cases of persistent microcytosis without apparent cause, it is essential to apply complementary molecular studies such as MLPA and NGS [24,25,28]. This strategy is key both for individual diagnosis and for genetic counseling of at-risk couples, in order to prevent severe cases such as hydrops fetalis [15,18,33]. Furthermore, the description and characterization of these new deletions directly contributes to the global catalog of structural variants of the α-globin cluster, helping to improve molecular and clinical recognition of these alterations in the contexts of prenatal diagnosis, blood donation, and population screening programs [4,5,37]. The variants described in this study have been submitted to both HbVar and IthaGenes databases (submission ID pending), to facilitate future clinical reference and genetic counseling.

### 3.1. Clinical Implications and Genetic Counseling

The identification of these novel α^0^-thalassemia deletions has important implications for clinical practice and genetic counseling. In the heterozygous state, carriers are usually asymptomatic, presenting only with microcytosis and hypochromia [13,18]. However, when combined with another α^0^-thalassemia allele or a non-deletional α^+^-thalassemia variant, these deletions can result in HbH disease, characterized by hemolytic anemia of variable severity [13,14,20]. More importantly, if both parents carry an α^0^-thalassemia allele, there is a 25% risk of conceiving a fetus affected by hemoglobin Bart’s hydrops fetalis, a condition that is typically lethal in utero or shortly after birth unless diagnosed prenatally and managed with advanced interventions [15,16,17,19].

In Spain, Hb Bart’s hydrops fetalis is extremely rare, with only isolated cases reported in national registries, and none documented in our center in the last decade [11,12]. Hemoglobin H disease shows highly variable clinical expression, ranging from mild, non-transfusion-dependent anemia to more severe phenotypes requiring intermittent transfusions, as described in previous studies [13,14,20].

For this reason, identifying rare and atypical deletions is crucial for accurate reproductive risk assessment [11,12,18]. Carrier detection and counseling allow for at-risk couples to be informed about potential outcomes and to consider reproductive options such as preimplantation genetic diagnosis or prenatal testing by chorionic villus sampling or amniocentesis [26,33]. In populations with Mediterranean ancestry, or in regions with increasing migration from endemic countries, the clinical value of early genetic counseling is particularly relevant [11,12,18].

Although family studies were proposed, only the index patients were analyzed in this report due to limited availability of relatives and consent constraints. Future studies including parental and offspring testing will be important to further characterize inheritance patterns.

Moreover, the incorporation of advanced molecular diagnostics, including MLPA and NGS, into prenatal and carrier screening programs improves the detection of atypical deletions that frequently escape conventional assays [24,25,26,28,44]. Expanded screening strategies are therefore essential to prevent severe outcomes such as hydrops fetalis and to provide comprehensive risk assessment for couples. Finally, the characterization and submission of novel deletions to databases such as HbVar and IthaGenes contribute to the global catalog of α-globin variants, facilitating more accurate counseling and supporting the development of evidence-based population screening programs [4,5,37].

### 3.2. Functional Relevance of HS-40 and Related Regulatory Mutations

The three deletions described here include loss of the HS-40 (MCS-R2) enhancer, which plays a central role in coordinating expression of the α-globin genes. Deletion of HS-40 alone has been shown to result in transcriptional silencing, even when HBA1 and HBA2 genes remain intact [28,30,32]. This effect is functionally equivalent to full structural deletions, and highlights the regulatory complexity of the α-globin cluster [31,38].

Interestingly, non-deletional point mutations in regulatory regions, such as the c.-59C>T variant in the HBA2 promoter, have also been associated with thalassemic phenotypes by disrupting transcription factor binding [7,27]. Comparative studies in erythroid cell lines and CRISPR-edited models have demonstrated that regulatory mutations can result in epigenetic silencing, altered chromatin architecture, and reduced α-chain synthesis [29,39,45]. These insights emphasize the need to functionally characterize non-coding and regulatory elements in cases of unexplained thalassemia phenotypes.

#### Limitations and Future Perspectives

This study presents some important limitations. The sample size is limited, and the data come from a single reference center, which hinders extrapolation of the true frequency of these deletions in the general population. However, the detection of three atypical variants supports the hypothesis of underdiagnosis of this structural mutation [12,26]. Although the genomic extent of the deletions has been precisely characterized thanks to MLPA and NGS, no functional studies in erythroid cells have been performed.

Future research should apply advanced technologies such as whole genome sequencing (WGS) to simultaneously detect structural and point variants, improving diagnostic coverage and efficiency [29,46]. Moreover, artificial intelligence (AI) algorithms trained on genomic and clinical data may enhance interpretation of complex or rare CNVs, prioritizing variants with pathogenic potential [47].

Finally, international efforts should aim to establish consensus guidelines for the classification, nomenclature, and reporting of novel large deletions in the α-globin cluster. Harmonized variant databases and publicly accessible reference materials will be crucial to support accurate diagnosis, cross-laboratory reproducibility, and equitable access to genetic information worldwide [5,6,44].

## 4. Materials and Methods

Three unrelated patients aged between 3 and 51 years were studied, referred to the Hematology Department of Hospital Clínico San Carlos in Madrid, Erythropathology Section, a reference center for the diagnosis of hemoglobinopathies and thalassemias. All cases were referred for persistent microcytosis not associated with iron deficiency, with initial hemoglobin studies showing no common structural variants.

Patient 1 was a 51-year-old male from Burgos, monitored for chronic microcytosis detected in routine tests, with no known family history or relevant clinical symptoms. Patient 2 was a 10-year-old girl from Santander. During a routine health check, part of the pediatric schedule for that age group, microcytosis and hypochromia were observed without iron deficiency or clinical signs of anemia. Patient 3 was a 3-year-old boy from Madrid, evaluated after microcytosis and hypochromia were detected during a child wellness check-up. His father was reportedly a thalassemia carrier, though he had not been genetically tested and was not available for analysis.

Hematological data were obtained using a Coulter DxH 900 hematology analyzer (Beckman Coulter, Brea, CA, USA). Hemoglobin fraction analysis was performed by two complementary methods: High-Performance Liquid Chromatography (HPLC) with the Variant II system (Bio-Rad, Hercules, CA, USA, Beta-Thalassemia Short Program), and capillary electrophoresis using the Capillarys Flex Piercing system (Sebia, Lisses, France). Although either method may be sufficient to detect hemoglobin variants, in our center, both are routinely performed to ensure diagnostic accuracy and cross-validation, particularly in pediatric cases.

Genomic DNA was extracted from peripheral blood using the BioRobot EZ1 automated system (Qiagen GmbH, Hilden, Germany), following the manufacturer’s protocol. DNA quantification was performed via fluorometry using the Qubit system (Thermo Fisher Scientific, Waltham, MA, USA) with the Qubit dsDNA BR Assay kit.

The molecular study followed a stepwise strategy for HBA locus characterization, starting with the most common variants and proceeding to full structural analysis: 1. Screening for frequent deletions was conducted using the ViennaLab StripAssay^®^ Alpha-Globin kit (ViennaLab Diagnostics GmbH, Vienna, Austria), which includes the most globally prevalent deletions (--SEA, --MED, --FIL, etc.). 2. Since the screening yielded negative results, Sanger sequencing of the exons and flanking regions of HBA1 and HBA2 was performed (BigDye Terminator v3.1 Cycle Sequencing Kit, Applied Biosystems, Foster City, CA, USA; capillary electrophoresis on 3130 Genetic Analyzer, Applied Biosystems, Foster City, CA, USA). No pathogenic point mutations or small insertions/deletions were found. 3. Multiplex Ligation-dependent Probe Amplification (MLPA) was then carried out using the SALSA MLPA P140 HBA kit (MRC-Holland, Amsterdam, The Netherlands) for complete structural analysis of the α-globin cluster. This technique revealed the loss of multiple consecutive probes, suggesting the presence of large deletions.

As part of the validation and expansion of our diagnostic capabilities, our lab recently incorporated Next-Generation Sequencing (NGS) with the specific Devyser Thalassemia panel (Devyser AB, Stockholm, Sweden). Retrospective NGS analysis of the samples was then performed to confirm and precisely delineate the deletions identified by MLPA. Bioinformatic analysis followed the manufacturer’s recommendations (Devyser Thalassemia Software, version 3.8, Devyser AB, Stockholm, Sweden).

## 5. Conclusions

This study expands the mutational landscape of α^0^-thalassemia by describing three novel large deletions encompassing HBA1, HBA2, and the HS-40 enhancer region. These findings reinforce the need for comprehensive molecular diagnostics in cases of unexplained microcytosis, particularly in non-endemic populations where rare deletions may go unrecognized. The combined use of MLPA and targeted NGS proved highly effective for the detection and genomic characterization of these complex structural variants. Their inclusion in public databases such as HbVar and IthaGenes contributes to international variant catalogs and supports clinical recognition, reproductive counseling, and population-level screening strategies. Future implementation of whole genome sequencing and AI-assisted variant interpretation could further enhance diagnostic precision and personalized care in hemoglobinopathies.

## Figures and Tables

**Table 1:** Hematological and hemoglobin data.

Patient	Sex/Year	RBC (10^6^/µL)	Hb (g/dL)	MCV (fL)	MCH (pg)	RDW (%)	HbA2 (%) HPLC	HbF (%) HPLC	Hb A2 (%) C.E
1	Male/51	7.0	15.5	69.0	18.0	15.3	2.5	<0.5	2.1
2	Female/10	6.3	12.4	63.0	19.6	15.6	2.9	0.6	2.3
3	Male/3	6.1	10.8	57.7	17.7	20.2	2.7	0.7	2.4

Red Blood Cells (RBC), Mean Corpuscular Volume (MCV), Mean Corpuscular Hb (MCH), Red Cell Distribution Width (RDW), and Capillary Electrophoresis (C.E). Reference ranges (age-adjusted): adults—MCV: 80–100 fL; MCH: 27–31 pg; MCHC: 32–36 g/dL. Children (4–7 years)—MCV: ~78–94 fL; MCH: ~23–31 pg; MCHC: ~32–36 g/dL. Infants (0–12 mo)—MCV: 70–123 fL; MCH: 23–37 pg. Reference ranges: age-adjusted. HbA2: 2.5–3.5% (adults); HbF: <1% in adults; sensitivity threshold of HPLC and capillary electrophoresis: 0.5%.

**Table 2:** Genomic characterization of the new α^0^-thalassemia deletions.

Patient	Deletion (MLPA Probe P140 HBA)	Estimated Size	Affected Region (Genes and Elements)	Type of Deletion	NGS Confirmation
1	Probes 1–30	~212 kb	*HBZ*, *HBA2*, *HBA1*, HS-40	Heterozygous	Yes
2	Probes 1–33	~360 kb	*HBZ*, *HBA2*, *HBA1*, HS-40	Heterozygous	Yes
3	Probes 1–29	~17 kb	*HBZ*, *HBA2*, *HBA1*, HS-40	Heterozygous	Yes

## Data Availability

The data supporting the findings of this study, including the identified genomic deletions, have been submitted to the HbVar and IthaGenes databases (submission ID pending). Additional anonymized clinical and molecular data are available from the corresponding author upon reasonable request.

## Abbreviations

The following abbreviations are used in this manuscript:

NGS	Next-Generation Sequencing
HPLC	High-Performance Liquid Chromatography
CE	Capillary Electrophoresis
MLPA	Multiplex Ligation-dependent Probe Amplification
